# Diversity of Culture Microorganisms from Portuguese Sweet Cherries

**DOI:** 10.3390/life13122323

**Published:** 2023-12-11

**Authors:** Ana C. Gonçalves, Amílcar Falcão, Gilberto Alves, Luís R. Silva, José D. Flores-Félix

**Affiliations:** 1 CICS–UBI—Health Sciences Research Centre, University of Beira Interior, 6201-506 Covilhã, Portugal; gilberto@fcsaude.ubi.pt (G.A.); luisfarmacognosia@gmail.com (L.R.S.); 2 CIBIT—Coimbra Institute for Biomedical Imaging and Translational Research, University of Coimbra, 3000-548 Coimbra, Portugal; acfalcao@ff.uc.pt; 3 Laboratory of Pharmacology, Faculty of Pharmacy, University of Coimbra, 3000-548 Coimbra, Portugal; 4 CPIRN-UDI/IPG—Centro de Potencial e Inovação em Recursos Naturais, Unidade de Investigação para o Desenvolvimento do Interior do Instituto Politécnico da Guarda, 6300-559 Guarda, Portugal; 5 Chemical Process Engineering and Forest Products Research Centre, Department of Chemical Engineering, Pólo II—Pinhal de Marrocos, University of Coimbra, 3030-790 Coimbra, Portugal; 6 Microbiology and Genetics Department, University of Salamanca, 37007 Salamanca, Spain

**Keywords:** sweet cherries, microorganisms, culturome, bacteria, fungi

## Abstract

Consumers today seek safe functional foods with proven health-promoting properties. Current evidence shows that a healthy diet can effectively alleviate oxidative stress levels and reduce inflammatory markers, thereby preventing the occurrence of many types of cancer, hypertension, and cardiovascular and neurological pathologies. Nevertheless, as fruits and vegetables are mainly consumed fresh, they can serve as vectors for the transmission of pathogenic microorganisms associated with various disease outbreaks. As a result, there has been a surge in interest in the microbiome of fruits and vegetables. Therefore, given the growing interest in sweet cherries, and since their microbial communities have been largely ignored, the primary purpose of this study is to investigate their culturome at various maturity stages for the first time. A total of 55 microorganisms were isolated from sweet cherry fruit, comprising 23 bacteria and 32 fungi species. Subsequently, the selected isolates were molecularly identified by amplifying the 16S rRNA gene and ITS region. Furthermore, it was observed that the communities became more diverse as the fruit matured. The most abundant taxa included *Pseudomonas* and *Ralstonia* among the bacteria, and *Metschnikowia*, *Aureobasidium*, and *Hanseniaspora* among the fungi.

## 1. Introduction

Nowadays, consumers seek safe functional foods with recognized health benefits that can offset oxidative stress and inflammatory processes, thereby preventing or ameliorating the occurrence of many diseases, including cancer, metabolic abnormalities, and neurological pathologies [1,2]. Therefore, given that fruits and vegetables contain numerous secondary metabolites, notably phenolics and terpenes, whose health-promoting potential is well-described, it is not surprising that their consumption has been increasing worldwide [3,4]. These metabolites are synthesized from primary metabolites, including organic acids, amino acids, and sugars, which in turn, are also essential to microorganism development [5]. In fact, an abundant and diverse community of microorganisms, especially bacteria, is found on the surface of the above-ground plant parts, which are collectively known as the phyllosphere [6]. The phyllosphere has a typical cell density of 10^6^–10^7^ cells cm^−2^ and is commonly subdivided into the caulosphere (stems), phylloplane (leaves), anthosphere (flowers), and carposphere (fruit) [6,7]. Although the microbiome can enhance the benefits of eating plants, namely by acting as probiotic agents, it is important to remember that most fruits and vegetables are commonly consumed raw without any processing or thorough washing, and therefore, they can serve as vectors for the transmission of pathogenic microorganisms (e.g., *Escherichia coli*, *Listeria monocytogenes*, *Salmonella* sp.) that are associated with various disease outbreaks that have been increasing over the years [8,9]. Indeed, it is estimated that one in every ten individuals in the world (an estimated 600 million people) falls ill after ingesting contaminated food, with 420,000 people dying each year [10]. Furthermore, microbiome communities can also affect a product’s economic value and reduce its nutritional and organoleptic properties [11,12,13]. As far as we know, microbiome communities are mainly influenced by cultivar genotype, maturation stage, and cropping practices [14,15,16,17].

Consequently, investigating the microbiome of foods is critical for food safety and consumption, as well as for preservation and growth control, and for contributing to the discovery of novel sources of beneficial bacteria and bioactive metabolites [18]. Sweet cherries (*Prunus avium* Linnaeus), which are diploid (2n = 16), are perishable and delicate berries with vibrant color, sweet taste, and pleasant aroma, which have been a target of recent interest in scientific research due to their richness in minerals, vitamins, and phenolics and their high water content [19,20,21,22,23]. Their popularity has grown as scientific data have demonstrated clinical evidence of their ability to relieve oxidative stress, reduce blood pressure and inflammatory markers, and improve aging and sleep [23,24,25,26,27,28]. Consequently, their incorporation in new supplements, nutraceuticals, and cosmetics is expanding worldwide (40% rise since 2000) [28,29]. These fruits are native to southern Asia and Europe, belonging to the *Rosaceae* family, *Prunoideae* subfamily, Prunus genus, which has about 430 species, and *Cerasus* and *Padus* subgenera [30]. They are deciduous trees, from 15 to 32 m in height, and with a trunk up to 1.5 m in circumference [31].

Furthermore, the analysis of communities associated with edible vegetables has been the subject of study due to their health implications, as they can act as vectors of diseases or influence the development and quality of the vegetables [32]. In the case of cherries, research has primarily focused on studying microbial communities under infection conditions that affect fruit quality, although interannual variation in surface populations has been studied [33,34]. For the Summit and Jiahong cultivars, the distribution and composition of bacterial communities follow a pattern similar to that observed in other plants, with high diversity in the root systems decreasing as we move away from them [35]. However, the composition of fruit microbiota remains poorly understood. Regarding fungal communities, there appears to be high variability, influenced by environmental factors and ascomycetes dominance [36].

Studies on microbial populations inhabiting fruits such as cherries have, therefore, garnered attention due to factors related to these fruits’ commercialization, such as extending the shelf life or post-processing procedures. These communities play a significant role in maintaining the fruit under optimal conditions, and the balance between saprophytes and pathogens may be essential for the fruit’s proper preservation [37]. Furthermore, certain yeast species, such as *Metschnikowia pulcherrima* or *Pichia kudriavzevii*, have demonstrated excellent biotechnological potential due to their antagonistic role against saprophytic microorganisms that could alter the product during long-distance transportation [38].

With these facts in mind, and with the knowledge that sweet cherries’ microbial communities are understudied, the main goal of the present work is to investigate their culturome at different maturation stages for the first time. Consequently, the selected isolates were molecularly identified via amplification of the 16S rRNA gene.

## 2. Materials and Methods

### 2.1. Chemicals and Reagents

All chemicals utilized were of analytical grade, and standard compounds were obtained from various suppliers. A Milli-Q water purification system (Millipore Ibérica, S.A.U., Madrid, Spain) was used to deionize the water (Millipore Ibérica, S.A.U., Madrid, Spain).

### 2.2. Sample Collecting

**Figure 1:**
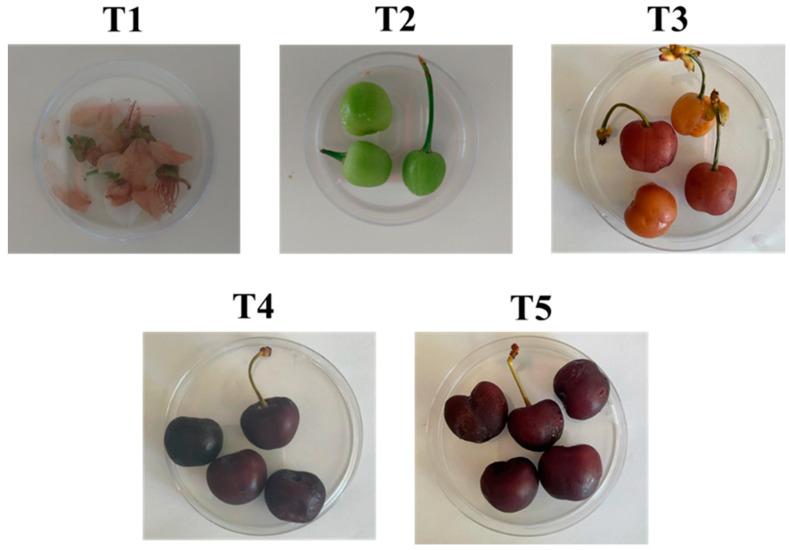
Cherry samples used in this study at different ripening stages.

Sweet cherry fruits from the cultivar Saco, widely cultivated in the Beira region, were supplied by a producer located in the Fundão region, Portugal (40°6′54.655″ N 7°36′26.701″ W). In 2021, samples were taken five times during their development on the mornings of the days 5 April (T1), 20 April (T2), 6 May (T3), 26 May (T4), and 7 June (T5) (Figure 1), from trees aged between 5 and 7 years old, their optimum productive stage. Samples were collected from five different trees, taking 10 cherries per tree and mixing them together. The harvesting was undertaken aseptically, using gloves and immediately placing the fruit into sterile plastic tubes. Then, the samples were promptly transferred to the laboratory facilities for processing at low temperatures. Transport was performed at 4 °C in an isothermic receptacle to ensure the appropriate refrigeration of the samples. Five to ten cherries were picked to study the microorganism culturome present at each stage of maturation.

### 2.3. Isolation, Cultivation, and Conservation of Microorganisms

The isolation of microorganism communities present in Portuguese cherries was performed using Sabouraud dextrose agar (SDA) with chloramphenicol for fungus, MRS agar, potato dextrose agar (PDA), and tryptic soya agar (TSA) for bacteria, and Reasoner’s 2A agar (R2A) medium for the possible growth of both microorganisms, and it was performed according to Gonçalves et al. [39]. To find out what microorganisms live in cherry tissues, collected samples were first washed three times with sterile deionized water and then surface-sterilized through immersion in 70% ethanol for 30 s and 2% sodium hypochlorite for 3 min. Finally, they were rinsed five times with sterile deionized water and sonicated for 2 min at 25 °C. Ten grams of superficially sterilized cherries were macerated under axenic conditions until achieving a homogeneous mass, and then resuspended in 90 mL of sterile distilled water. The mixtures were stirred for one hour at 125 rpm. The samples were then serially diluted and seeded in the appropriate media. Then, Petri dishes were incubated at 22 °C and 28 °C to encourage possible fungal and bacterial growth, respectively, and the formation of colonies was monitored for four days. Two sterilization controls were employed. In the first one, entire surface sterilized fruits were placed in Petri dishes to ensure that isolates were endophytes, while in the second one, 100 µL of water employed for sonication in the last step of sterilization was spread in TSA and SDA media. Growth controls were checked after 120 h to ensure the absence of colony development. At the conclusion of this period, the microorganisms were isolated based on their phenotypic characteristics and again cultivated to grow. This last procedure was repeated several times until pure cultures were obtained. Finally, pure cultures were preserved at −80 °C in glycerol.

### 2.4. Infraspecific Diversity Analysis and Identification Based on 16S rRNA Sequencing

Total DNA extraction was performed using the NZY Plant/Fungi gDNA Isolation Kit (NZY Tech, Lisbon, Portugal) according to the manufacturer’s instructions to identify known microorganisms present in the cherry samples. The genetic diversity of isolated strains was assessed using RAPD fingerprinting performed, as previously described [40], with the primer M13 (5′-GAGGGTGGCGGTTCT-3′) and the Dream-Taq™ DNA Green PCR Master Mix (Fisher Scientific, Waltham, MA, USA). PCR conditions were the following: preheating at 95 °C for 9 min, 35 cycles of denaturing at 95 °C for 1 min, annealing at 45 °C for 1 min, and extension at 75 °C for 2 min, with a final extension at 72 °C for 7 min. Aliquots of 17 µL of each PCR product were electrophoresed on 1.5% (*w*/*v*) agarose gel in TBE buffer (100 mM Tris, 83 mM boric acid, 1 mM EDTA, pH 8.5) at 6 V/cm. The gels were stained in a solution containing 0.5 mg/L ethidium bromide, and photographed under UV light. Standard VI (Roche, Basilea, Switzerland) was used as a size marker. A dendrogram was constructed based on the matrix generated using the UPGMA method and Pearson’s coefficient with Bionumerics version 4.0 (Applied Maths, Austin, TX, USA). For RAPD group delimitation, we applied a threshold of 70% similarity.

The amplification and sequencing of the 16S rRNA genes were carried out, as indicated previously [39], using the primers 27F (5′-AGAGTTTGATCCTGGCTCAG-3′) and 1522R (5′-AAGGAGGTGATCCANCCRCA-3′). The acquired sequences were compared to those in GenBank using the BLASTN program, selecting “type strains” as the phylum to perform the search [29]. The sequences obtained were deposited in GenBank. Table 1 includes accession numbers of the representant strain from each RAPD group.

## 3. Results and Discussion

As previously mentioned, investigating bacterial and fungal epiphytes and endophytes is valuable and essential, as both communities have a significant impact on food development, organoleptic characteristics, and nutritional value. Indeed, these assessments may be regarded as providing baseline data for the discovery of new pharmaceutical and therapeutic molecules, as well as to address wider ecological issues [5].

**Figure 2:**
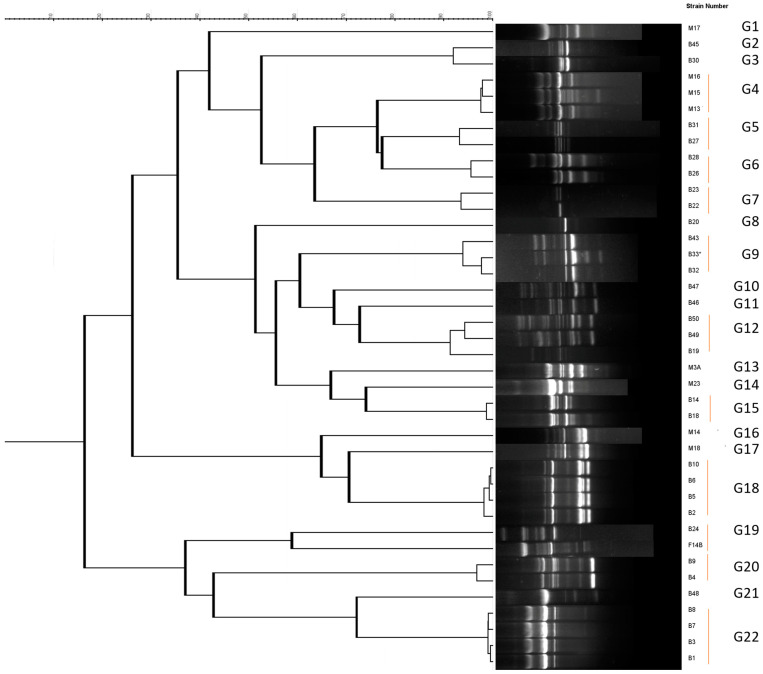
Dendrogram obtained for the bacterial strains isolated in the present study using Pearson’s coefficient and UPGMA (Unweighted Pair Group Method with Arithmetic Mean) analysis of the RAPD profiles.

**Figure 3:**
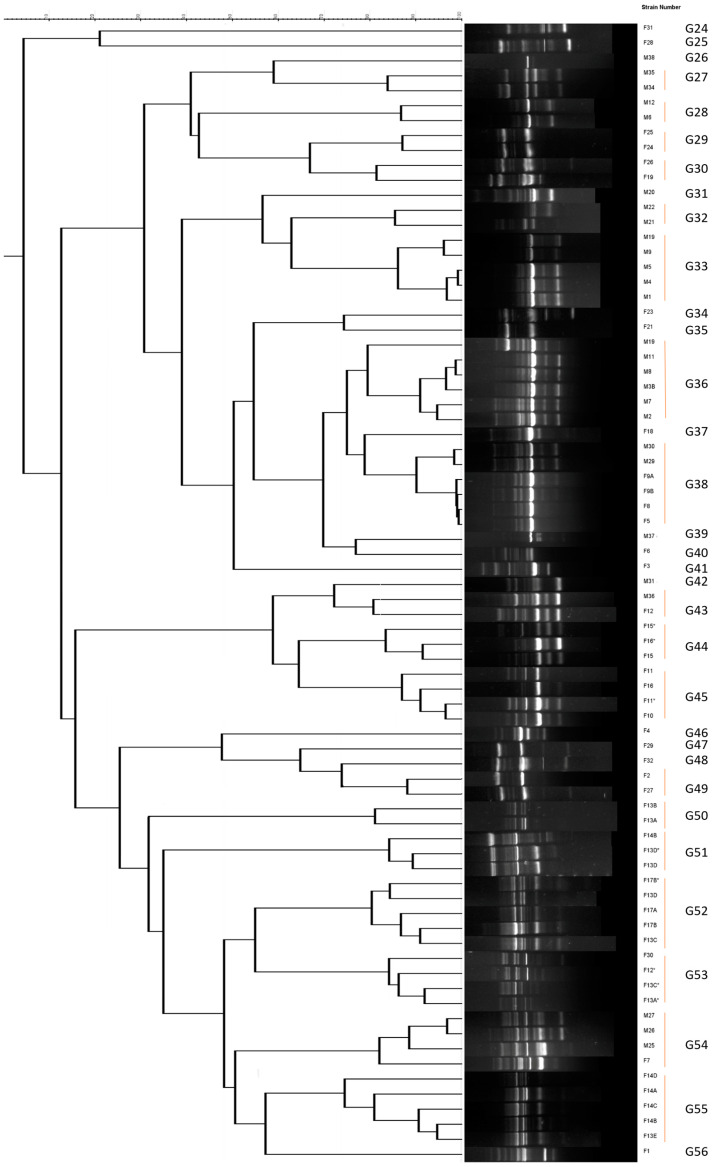
Dendrogram obtained for the fungal strains isolated in the present study using Pearson’s coefficient and UPGMA (Unweighted Pair Group Method with Arithmetic Mean) analysis of the RAPD profiles.

**Figure 4:**
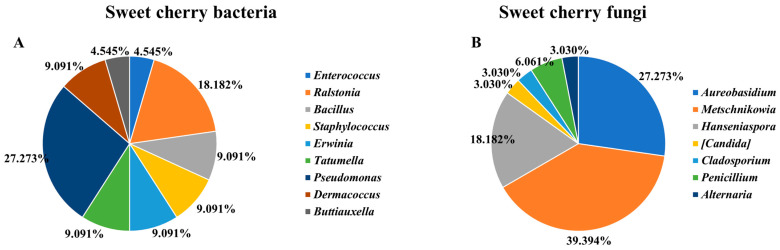
Most representative bacteria (**A**) and fungi (**B**) found in sweet cherry fruit.

In the current investigation, a total of 55 microorganisms, including 23 bacteria and 32 fungi, were isolated from sweet cherry fruits over a period of approximately 9 weeks, as described in Table 1 and Figure 2, Figure 3 and Figure 4. In particular, a total of 125 × 10^3^ UFC/g bacteria and 212 × 10^2^ UFC/g fungi were found on TSA and SDA media, respectively. Therefore, several different strains with a relatively significant infraspecific diversity were isolated, with a higher proportion of fungi than bacteria (Figure 2 and Figure 3). As previously mentioned, this is the first study of the sweet cherry culturome. The procedure was designed to isolate the maximum possible quantity of microorganisms present during the development of the current fruit.

Focusing on bacteria, fingerprints RAPD were grouped into 22 clusters or clades (Figure 4A), in accordance with the number of bands and molecular weights obtained using M13-RAPD, and a representant of each group was selected for sequencing. Genera *Pseudomonas* and *Ralstonia* were the most dominant strains, representing 26.09% and 21.74%, respectively, followed by *Bacillus*, *Staphylococcus*, *Erwinia*, *Tatumella*, *Dermacoccus*, and *Buttiauxella* (each 8.70%). *Enterococcus* was the least abundant (4.35%). The *Erwinia tasmaniensis*, *Pseudomonas viridiflava*, and *Paucimonas lemoignei* strains were only found in T1. On the other hand, *Pseudomonas edaphica* was isolated at T2 and remained at T3, while *Ralstonia pickettii* was present at the T3, T4, and T5 stages. *Enterococcus* and *Buttiauxella* were only detected at the end of the development (T5). The richest bacterial stages were T3 and T5. With regard to other red fruits, *Curtobacterium* (19.88%), *Pseudomonas* (15.06%), *Microbacterium* (13.86%), and *Clavibacter* (12.65%) are considered the representative genera found in plums, while *Enterobacter* (5.42%), *Chrysomonas* (4.82%), and *Pantoea* (4.22%) are less abundant. Among them, *Microbacterium* and *Curtobacterium* predominate in the early stages of fruit growth, while *Pseudomonas* and *Clavibacter* are predominant at the maturity stage [14]. Furthermore, Xu and colleagues [18] investigated the microbiome of mulberry fruits and identified 608 distinct endophytic bacteria, with *Proteobacteria* (62.83%), *Firmicutes* (26.81%), and *Actinobacteria* (9.87%) dominating, and the phyla *Bacteroidetes* (0.94%) being the least abundant. With regard to strawberries, *Actinobacteria*, *Alphaproteobacteria*, *Gammaproteobacteria*, *Deltaproteobacteria*, and *Bacteroidia* represent the most prevalent bacterial groups, accounting for about 80% [16]. *Curtobacterium* (21.31%) and *Pseudomonas* (19.99%) were the most frequent genera identified in nectarine fruits, followed by *Microbacterium* (13.57%), *Clavibacter* (9.69%), *Pantoea* (6.59%), and *Enterobacter* (4.26%) [15].

These studies highlight the significant diversity exhibited by plant tissues associated with fruits, especially epiphytic ones, which are highly influenced by the environmental and cultivation conditions to which the plant is subjected. Moreover, endophytic communities in fruits appear to have low diversity and high susceptibility to pathogen presence. Due to their biology, these pathogens efficiently colonize those tissues either internally or externally, such as through transportation by animal vectors. Some of the genera found, such as *Erwinia bilingae* or *Pseudomonas syringae*, may have a pathogenic nature towards the plant itself, although their presence may not be of great significance if the microbiota composition is balanced [41]. The well-known role of diverse microbiotas in controlling the action of pathogens underscores this point. On the other hand, the isolation of opportunistic human pathogens, such as *Ralstonia picketti* or *Staphylococcus* sp., is noteworthy, demonstrating that these microorganisms are present in foods. However, their impact is influenced by other factors such as the presence of pathogenicity genes, the number of individuals in the sample, the host’s condition, and susceptibility to disease [42].

On the other hand, *Erwinia tasmaniensis* and *Pseudomonas viridiflava* are epiphytic species that invade necrotic tissues, while also being used as antagonists for fire blight biocontrol [43,44,45]. Nonetheless, most *Pseudomonas* and *Dermacoccus* promote plant growth by suppressing pathogenic microorganisms and offering protection against climate change [46,47,48,49]. A *Dermacoccus nishinomiyaensis* strain, in particular, produces considerable amounts of indole-3-acetic acid, an auxin involved in seed germination, root formation, and embryo and fruit development [50]. The genus *Bacillus* is a broad genus that currently includes 109 valid species of bacteria, alongside a further 17 different genera recently described as independent, though they traditionally belonged to this genus [51]. The isolation of strains of this genus from plant tissues is reported as common in numerous studies, and they can be isolated from any plant tissue, although they are more abundant in rhizospheric and epiphytic environments [52]. Within *Bacillus* and other related genera, we found some species associated with plant development and protection, particularly after harvesting, conferring to plants a high resistance against disease and degradation [53]. The potential of this genus is widely recognized, as it encompasses bacteria with multiple desirable characteristics from a biotechnological perspective. The technological features of these bacteria, attributed to the formation of resistant spores and their broad metabolic capabilities, have led to numerous studies focusing on them [54]. Conversely, *P. lemoignei* is unique among poly(R)-3-hydroxyalkanoates-degrading bacteria, since it is capable of synthesizing at least six different extracellular poly(R)-3-hydroxyalkanoate depolymerases, and hence, it can be a useful tool in degrading plastics [55]. *Staphylococcus epidermidis*, which is largely found in meat products, has been linked to hospital infections [56]. *Staphylococcus warneri*, which is also found in apples, is a common commensal microorganism present in the skin microbiota of individuals who are highly resistant to penicillin. Although it rarely causes infections in healthy people, in immunosuppressed patients or individuals with cirrhosis, it can cause multiple subcutaneous abscesses or urinary infections, respectively [57,58]. *Buttiauxella ferragutiae*, which is also found in mulberries, is a novel species of *Kluyvera* that is associated with urinary tract infections in children [18,59]. Furthermore, clinical evidence has already been reported of a positive correlation between *Enterococcus* spp. presence and urinary tract infections [60]. *Tatumella ptyseos* is related to a food-borne opportunistic pathogenic microorganisms related to neonatal sepsis, urinary tract infections, and bacteraemia [61], whereas *Ralstonia pickettii* is found in the gut microbiota of individuals with metabolic disorders, and in mesothelioma patients [62,63].

In contrast, 34 RAPD groups were obtained for fungi, and 7 different genera of fungi were identified (Figure 4B). *Metschnikowia* sp. (40.63%) was the most common genera found during cherry development, followed by *Aureobasidium* (25.00%) and *Hanseniaspora* (18.75%). *Cladosporium* and *Alternaria* species, in particular, were only detected in the early stage of development, i.e., in T1. The *Aureobasidium pullulan* strain was detected in both T1 and T2 stages, while most *Metschnikowia* subclasses were present in T2. In contrast to bacteria, T3 was low in fungi development, with the only detected species being *Hanseniaspora uvarum* and *Hanseniaspora pseudoguilliermondii*. *Hanseniaspora pseudoguilliermondii* was also found in T4 and T5, while *Hanseniaspora pseudoguilliermondii* was again detected in T5. In addition to *Hanseniaspora pseudoguilliermondii*, *Aureobasidium pullulans* and *Candida oleophila* were the only other fungi found in T4. At the last stage of cherries’ development, i.e., in T5, nine different fungi were identified, with the presence of *Penicullium crustosum*, *Hanseniaspora uvarum*, and *Metschnikowia pulcherrima* strains standing out. A higher number of fungi in T5 is to be expected, contributing to the fermentation process. With regard to other fruits, *Mycosphaerella* (45%), followed by *Mortierellaceae* (11.3%) and *unidentified Capnodiales* (10.5%), are the fungal families most found in strawberries [64], while *Aureobasidium* (49.86%), *Alternaria* (18.43%), *Hanseniaspora* (17.63%), and *Pleospora* (6.63%) are commonly identified in grapes [65]. In apples, *Articulospora*, *Bullera*, *Cryptococcus*, *Dioszegia*, *Erythrobasidium*, and *Sporobolomyces* are the most dominant (49.5%), while Cladosporiaceae, Sclerotiniaceae, and Mycosphaerellaceae are highly abundant in blueberries, accounting for 40.9% of total fungi [66,67]. These data indicate that the composition of the fungal community associated with sweet cherries from the Beira Interior region in Portugal shares numerous common elements with other fungal communities in edible fruits. While it is known that plants have a significant capacity to select the taxa that form their microbiome [39,68], there may be a greater environmental influence on more exposed organs such as fruits or flowers. Alternatively, the specific conditions of these tissues may favor the development of similar taxa across different plant species.

*Cladosporium subuliforme* has been linked with fruit diseases [69]. However, [*Candida*] *olepphila*; *Aureobasidium* sp., particularly, *pullulans*; and *Metschnikowia* sp., such as *M. pulcherrima*, protect against postharvest fruit decay. This again applies particularly to *A. pullulans*, which is also involved in the production of volatile organic compounds [70,71,72,73]. Nonetheless, other authors highlight the ability of strains of the species *Aureobasidium pullulans* and *Metschnikowia pulcherrima*, isolated from the carposphere of *P. avium*, to control degradative processes mediated by microorganisms or plant pathogens such as *Sporobolomyces roseus* or *Cryptococcus wieringae*. Additionally, they show significant antagonistic activity against strains of their own genus and others, such as *Saccharomyces cerevisiae* [36]. These data indicate that the activity depends on the specific strain studied and the dominance of a particular strain among populations. The strains studied are derived from fruits in a perfect condition, showing no signs of rot or degradation and thus being in an optimal state for consumption. However, *Hanseniaspora uvarum* also produces volatile compounds, that contribute to fruits’ flavor and defense during storage at cold temperatures [74], while *H. pseudoguilliermondii* degrades organic acids and, consequently, reduces acidity [75]. Together with *H. meyeri*, both have already shown potential to produce amylases, pectinases, cellulases, and proteases [76]. *Metschnikowia sinensis* is related to a cider aroma [77]. *Alternaria* sp. are invasive fungi that infect fruits and vegetables and produce *Alternaria* toxins resulting in deleterious effects on human health, such as damaging the heart and lungs [78], while *Penicillium crustosum* produces mycotoxins associated with fruit blue mold decay, principally of apples [79]. However, the presence of strains belonging to the genus *Penicillium*, and also strains of the species *P. crustosum*, is common in fruit samples, especially in mature samples. They become particularly abundant when degradation processes commence due to the saprophytic nature of this genus [80].

Finally, the current study offers further evidence that the fermentative yeasts *Hanseniaspora* and *Metschnikowia* are typically found in the later phases of development.

## 4. Conclusions

Given the current consumption of fruits and vegetables, it is crucial to investigate their culturome, since most of them are commonly consumed fresh and may be potential vectors of food-borne pathogen diseases. In addition, their microorganisms may be regarded as a rich reservoir of bioactive compounds that influence foods’ quality, characteristics, and nutritional value and exert positive effects on human health. Although sweet cherries are highly appreciated by consumers, there has been a lack of analysis with regard to their culturome. The present study has, however, achieved the identification of 23 bacterial and 25 fungal strains. With regard to bacteria, *Ralstonia* and *Pseudomonas* were the most dominant, each representing around 21.74% of the total, followed by *Bacillus*, *Staphylococcus*, *Erwinia*, *Tatumella*, and *Dermacoccus* (each 8.70%). With regard to fungi, *Metschnikowia* sp. (44.00%) is the most abundant genera, followed by *Hanseniaspora* (20.00%) and *Aureobasidium* (16.00%). The microbial community of cherries from different sources and environments, and related fruit produce, need further investigation, as do the interactions between microbial species, to ensure their safety and increase their economic value.

## Figures and Tables

**Table 1:** Culturome of sweet cherry cultivars in different maturity stages. The presence of each strain in different times of development was marked with an “x”.

Strain	RAPD Group	Identity	% Identity	Accession Number	T1	T2	T3	T4	T5
Bacteria									
B2	G18	*Erwinia tasmaniensis*	99.19	OR517226	x				
B4	G20	*Pseudomonas viridiflava* strain	98.90	OR517224	x				
B7	G22	*Pseudomonas syringae*	99.57	OR517209	x				
B18	G15	*Pseudomonas edaphica*	99.93	OR517218		x	x		
M18	G17	*Dermacoccus nishinomiyaensis*	100	OR517219			x		
B20	G8	*Pseudomonas trivialis*	99.34	OR517220			x		
B22	G7	*Staphylococcus epidermidis*	99.59	OR517212			x		
B24	G19	*Staphylococcus pasteuri*	100	OR517214			x		
B28	G6	*Erwinia billingiae*	99.54	OR517221			x		
B30	G3	*Bacillus aerius*	98.70	OR517228			x		
B27	G5	*Ralstonia pickettii*	99.53	OR517208			x		
B33	G9	*Ralstonia pickettii*	99.92	OR517210			x	x	x
B45	G2	*Bacillus altitudinis*	99.86	OR517211					x
B46	G11	*Enterococcus rotai*	99.34	OR517225					x
B48	G21	*Tatumella terrea*	98.47	OR517213					x
B49	G12	*Ralstonia pickettii*	99.33	OR517222					x
B47	G10	*Ralstonia pickettii*	100	OR517223					x
M17	G1	*Pseudomonas qingdaonensis*	98.69	OR517215					x
M23	G14	*Tatumella ptyseos*	99.79	OR517216					x
M16	G4	*Pseudomonas graminis*	99.80	OR517217					x
M14	G16	*Dermacoccus nishinomiyaensis*	98.42	OR517227					x
M3A	G14	*Buttiauxella ferragutiae*	99.80	OR517230					x
Fungus									
F1	G56	*Cladosporium subuliforme*	99.80	OR584279	x				
F2	G49	*Aureobasidium pullulans*	100	OR584267	x				
F21	G35	*Metschnikowia pulcherrima*	100	OR584285	x				
F8	G38	*Aureobasidium pullulans*	95.76	OR584276	x				
F4	G46	*Aureobasidum proteans*	99.43	OR584284	x				
F6	G40	*Hanseniaspora uvarum*	98.86	OR584286	x				
F7	G54	*Alternaria conjuncta*	99.53	OR584275	x				
F10	G45	*Metschnikowia pulcherrima*	100	OR584266		x			
F29	G47	*Metschnikowia pulcherrima*	100	OR584265		x			
F12	G43	*Metschnikowia ziziphicola*	97.02	OR584271		x			
F23	G34	*Metschnikowia pulcherrima*	100	OR584294		x			
F13A	G50	*Metschnikowia chrysoperlae*	100	OR584277		x			
F13D*	G51	*Metschnikowia pulcherrima*	97.50	OR584274		x			
F14A	G55	*Metschnikowia pulcherrima*	99.73	OR584278		x			
F15	G44	*Aureobasidium pullulans*	100	OR584293		x			
F13C	G52	*Metschnikowia sinensis*	98.78	OR584268		x			
F18	G37	*Aureobasidium pullulans*	99.38	OR584262				x	
F19	G30	[*Candida*] *oleophila*	99.62	OR584273				x	
F25	G29	*Hanseniaspora pseudoguilliermondii*	99.04	OR584290				x	
F28	G25	*Metschnikowia pulcherrima*	97.64	OR584287				x	
F30	G53	*Hanseniaspora uvarum*	99.72	OR584264			x		x
F31	G24	*Metschnikowia pulcherrima*	97.64	OR584263					x
F32	G48	*Hanseniaspora pseudoguilliermondii*	99.04	OR584272			x	x	x
M20	G31	*Metschnikowia pulcherrima*	98.85	OR584269					x
M38	G26	*Hanseniaspora uvarum*	99.46	OR584270					x
M22	G32	*Penicillium crustosum*	100	OR584280					x
M37	G39	*Penicillium crustosum*	100	OR584281					x
M31	G42	*Hanseniaspora meyeri*	100	OR584282					x
M34	G27	*Metschnikowia pulcherrima*	99.12	OR584283					x
M31	G42	*Aureobasidium proteae*	95.71	OR584288					x
M6	G28	*Aureobasidium pullulans*	95.76	OR584289					x
M4	G33	*Aureobasidium pullulans*	95.76	OR584291					x
M2	G36	*Aureobasidium pullulans*	95.76	OR584292					x

## Data Availability

Data presented in this study are available within the article.
